# The Causes of Transient Cerebral Paralyses

**Published:** 1909-03

**Authors:** George Parker

**Affiliations:** Physician to the Bristol General Hospital


### THE CAUSES OF TRANSIENT CEREBRAL PARALYSES.

BY

GEORGE PARKER, M.A., M.D. Cantab.,
*Physician to the Bristol General Hospital.*

---

THE prevention of what are commonly called "apoplectic strokes" is one of the most important and most interesting fields for research at the present time. They cut short so many vigorous lives, or reduce them almost to the level of a vegetative existence. They arise, too, in the course of so many different diseases, and checkmate us when a prospect of recovery is in sight.

The majority of the cases are accompanied by organic lesions, and offer little hope of repair beyond the substitution and training of other nerves and muscles. Certain hemorrhages, indeed, may be absorbed, and if important structures have not been damaged ultimate recovery may take place. In embolism, again, the collateral vessels are sometimes able to restore the circulation, and the paralysis may then vanish entirely. Indeed, even in permanent cases the extent of the primary damage decreases when the immediate œdema passes away. In strictness too many poisons—such as narcotics and asphyxiants—produce temporary general paralysis and unconsciousness.

Apart from all these, there is a group of rare instances where no organic lesion is found, which are not due to poison, and where complete and often sudden restoration of function may take place. In some the recovery is permanent; in others recurrent attacks are seen which may eventually lead to organic changes and permanent paralysis.

I have recently met with some four or five of these transient paralyses, and a study of the literature shows a considerable number of similar ones. I propose, then, to consider how such attacks are brought about, without entering upon the large and debatable subject of the transient paralyses in certain diseases, such as hysteria and epilepsy, as to which we have very little knowledge. Uræmic paralyses seem worth discussion, however, as some light has been thrown on their pathology. One of my cases occurred in uræmia, two probably were due to arterio-sclerosis, one arose in a healthy man under great mental excitement with possible sclerosis, and another in gouty glycosuria. They all, even the uræmic and gouty ones, seem to be due to some form or other of local and temporary cerebral anæmia.

The following are brief notes of my patients :—

**Case 1.**—A.N., 44, male. Suffering from chronic nephritis, with six to eight grammes of albumen per litre, numerous casts, and thirty to forty ounces of urine daily. Began to show signs of uræmia by nausea and nocturnal dyspnœa. On October 26th, at 2 a.m., he was found to have a difficulty in speaking, and some weakness of the left side of the face and left arm. A slight convulsion followed. The paralysis increased next day. From October 29th to November 1st the whole of the left side was completely paralysed. The breathing was stertorous, the pulse very feeble. The patient was unconscious for some time, and even when conscious could not put out his tongue or open his mouth. Swallowing was very difficult. No optic neuritis was present. There was œdema of the left leg, arm and left side of trunk and head. On November 4th the paralysis had almost entirely gone, and the patient was rational and able to talk and swallow. The systolic blood pressure before the attack was 150 mm., but rose some months later on.

**Case 2.**—M., 71, female, had suffered for some years from gout and dyspepsia, and had recently recovered from an acute erythematous affection of one foot, with excoriation, and then a general dermatitis. On September 20th, while writing a letter, her sight suddenly became dim. For a few minutes she continued to write unmeaning phrases. She next found that she had lost her speech, and with some difficulty she made her way to her bed. When I saw her she was aphasic, and the sight and hearing were both affected. The right hand and foot were not paralysed, but the right side of the face was paretic. All the symptoms disappeared after three or four days. There had been no albumen in the water previously, but now a cloud of albumen and .4 per cent. of sugar were noted. She has remained in good condition ever since, except for some neuritis, chiefly in the right leg, and persistent dyspepsia. The sugar has gradually decreased. The blood pressure was about 135, and the pulse 76 to 80.

**Case 3.**—H.M., 69, male, who had been going through very great mental worry, was specially disturbed one evening over his business, and complained of feeling very strange. He slipped off his seat on to the floor, and gradually became unconscious, Clonic convulsions and twitching, especially on the right side. appeared, while the left was completely paralysed. The breathing was stertorous, and the corneal reflex lost. The attack lasted, perhaps, three hours. Next morning he had recovered perfectly, and showed no trace of the paralysis. The kidneys were normal. The blood pressure was about 140, and there was a slight blowing systolic murmur heard over the base of the heart. No arteriosclerosis was observed, and he has remained in good health ever since.

**Case 4.**—G.W., 61, male, had been recently undergoing very great trouble and worry, and when bending down one evening to wash was suddenly attacked with partial loss of power in the right arm and leg. He was admitted to the General Hospital the same evening (June 18th), when right hemiplegia, slight aphasia and paralysis of right side of face were noticed. The knee-jerk on the left side was exaggerated, and that on the right was normal. No Babinski reflex could be obtained. Urine, 1027, no albumen or sugar; blood pressure, 140; chest, emphysematous. Heart: apex in nipple line, sixth space; no murmur. June 22nd.—No paralysis anywhere, but patient complained of pains in the head. No anæsthesia; reflexes normal. Marked arcus senilis. Brachial artery thickens. The temperature varied between 97–99° F.

**Case 5.**—M.H.P., 70, female, had usually good health, but had at least once passed gall-stones. For the last five years of her life she suffered from occasional attacks of aphasia and vertigo, lasting for a few minutes, but without loss of consciousness. In June, 1907, after an indigestible meal, gastritis and acute vomiting prostrated her for three days. She seemed well for a day or two, but awoke one morning to find herself partially aphasic and hemiplegic. She could still grasp feebly with the affected hand, and stand when supported. The pulse was fairly strong and regular; the blood pressure 170. The paralysis gradually increased, and death took place in November. No kidney trouble existed, but arterio-sclerosis seemed general. Here slight transient attacks had recurred for years, to be finally replaced by a permanent one.

To return to the general question of the causes of transient cerebral paralyses. They can be produced by stoppage of the brain circulation in certain areas, or by anything which interferes with the physiological action of the blood; by poisons which directly affect the nervous substance; conceivably by direct pressure on the motor tract; or by exhaustion of certain nerve centres. We may provisionally, then, tabulate the causes as follows :—

(*a*) *Cerebral Anæmia or stasis*:

(1) General, produced by—

(i) Compression of the vessels of the neck.

(ii) Cardiac or respiratory failure.

(iii) General vaso-motor paresis.

(iv) Asphyxia by $CO_2$ and similar bodies.

(2) Local obstruction or compression of cerebral vessels from—

(i) Arterio-sclerosis or atheroma.

(ii) Syphilitic thickening.

(iii) Tumours and foreign bodies.

(iv) Effused blood and the œdema of softening.

(v) Uræmic œdema and hydrocephalus.

(vi) Inflammatory effusions, as in meningitis.

(vii) Certain small emboli.

(viii) Possibly spasm in Raynaud's disease and migraine.

(*b*) *The action of poisons, exhaustion of nerve centres, and unknown causes as seen in:*

(1) Narcotic poisoning.
(2) Hysteria.
(3) Eclampsia.
(4) General paralysis, myasthenia gravis.
(5) Post-epileptic states, some forms of petit mal.
(6) Chorea paralytica.
(7) Lead encephalopathy.
(8) Recurrent paralysis of the third nerve.

In the first group we have a number of causes which act on the hæmic stream. From the well-known experiments of Kussmaul, Tenner, and many others, we find that sudden occlusion of the arteries of the neck leads to convulsions, and then to paralysis. If the cerebrum alone be deprived of blood, we get paralysis and unconsciousness. If the bulb suffers too, convulsions. Similar results appear in the condition named after Stokes-Adams. Stoppage of the venous outflow has nearly the same effect, and so has a substitution of saline fluid for arterial blood. It is often said that " an increased flow of blood to the head " may cause like results, but in normal conditions " increased arterial flow is at once followed by increased venous outflow," *i.e.* the rate of the current alone is increased. The circulation in the brain under a rise of arterial pressure is converted into a system of rigid tubes, and there is practically no evidence that continued high pressure can cause paralysis or other cerebral symptoms.[1] If both veins and arteries are occluded, as in hanging, the results are the same as in compression of the arteries alone. People who have been hanged and cut down before death, describe their feelings as including, first a tingling in the limbs, then convulsions, and then complete loss of power to move them, before the blank of unconsciousness comes on.[2] Hence so many die in accidental hanging when a slight movement to seize some support would have saved them. Again, as Dr. Leonard Hill points out, the cerebral pressure varies absolutely with the venous pressure of the body, and only relatively with the arterial, and therefore venous obstruction alone may give rise to the symptoms of cerebral stasis or anæmia. Thus paralysis may occur in respiratory obstruction, or in the sinus thrombosis of chlorosis. I have seen a transient hemiplegia in whooping cough, though here the possibility of a small hemorrhage must not be forgotten.

Localised vascular obstructions are of still greater importance. It is a common experience that a tumour, foreign body, or depressed bone does by compression of veins and capillaries cause stasis in a given area and the symptoms of local anæmia, while a return of function follows the removal of the mass. Similar transient paralyses may follow the displacement of an embolus. Very much discussion has arisen as to whether local cerebral anæmia arises solely from such causes, or whether active spasmodic constriction of the cerebral vessels can take place. Not long ago Dr. Leonard Hill stated that trustworthy methods had as yet failed to demonstrate any action of vaso-motor nerves on these vessels, and it is certainly quite clear that they are not affected by the general vaso-motor centre. Gulland, indeed, showed the existence of nerves on the pial vessels, but no contractile power has been demonstrated in them by ordinary methods. Most observers have failed to produce contraction of the vessels in any degree, after trying all kinds of vaso-constrictors, such as the injection into them of suitable drugs to act on their muscular coats. On this view, the cerebral vessels passively receive and transmit the blood stream, the pressure of which is predetermined by the variations of the systemic, and especially of the splanchnic, vessels.

On the other hand, Bastian, W. E. Russell, and the Scotch school insist that while these brain vessels are not linked up to the general vaso-motor centre, they are still capable of local or general constriction from nerve stimuli, or from substances in the blood stream acting directly on their muscles, such as the products of the suprarenal and pituitary glands. Cushing[3] has been able to demonstrate the action of adrenalin in blanching the pial vessels, and claims that under some circumstances Faradic stimulation will contract the cortical vessels. Carl F. Wiggers,[4] too, has been able to show by special methods some active vaso-motor changes produced by drugs, and in a later paper, based on the pressure variations in tubes supplying an artificially-perfused brain, he gives further evidence of the action of vaso-motor nerves. Even under the most delicate tests, however, the action is slight, and the muscular coats are peculiarly feeble compared to those of the rest of the body. Corroborative evidence is found in certain cases of Raynaud's disease.[5] Thus Osler[6] reported the concurrence of hemiplegia and aphasia with a typical spastic condition of the vessels in a limb. Lindsay Steven[7] describes a similar concurrence where *post-mortem* an area of white necrosis without visible arterial disease was found in the brain area related to the paralysed limbs. Finally, if the retinal vessels are looked on as part of the cerebral circulation, it may be worth noticing that W. E. Russell and others[8] have seen a temporary contraction of the retinal arteries, both in Raynaud's disease and in quinine poisoning, while Cushing and Bordley point out that trephining not only relieves cerebral compression, but abolishes the existing retinal changes at the same time.[9] Still, on the whole I am inclined to think that, while we have good evidence of a slight degree of active vaso-motor power in the cerebral vessels in ordinary conditions, it is almost entirely overshadowed by the far greater and stronger systemic variations, to a much higher degree, perhaps, than the pulmonary vaso-motor agency is overshadowed by the splanchnic. Thus we are justified in most cases in regarding the cerebral circulation as a passive one, in spite of the researches of Wiggers and others, at the instance of Prof. Kocher. Again, too much weight should not be given to the striking phenomena in Raynaud's disease, for the contractions there, even in the systemic circulation, are of quite unknown pathology, of exceptional severity, and not capable of experimental reproduction. It is not reasonable to invoke the tempting hypothesis of a local spasm of the feeble cerebral vessels to explain every difficulty, since there is abundant evidence that ordinary vaso-constrictors entirely fail to produce it. Even if we add this additional link to the chain, we have still to explain what causes this spasm in the vessels of a certain isolated area. We are no better off, and it is simpler to suppose a toxin in the cells of that area at once.

*Arterio-sclerosis.*—How, then, can we explain the many cases of transient paralysis found in persons of perfect health except for sclerotic changes, local or general, in their vessels? Their blood pressure is often high, but sometimes the reverse. Now if certain vessels are partially occluded and inexpansile from sclerosis or cerebral syphilis, when any rise of general blood pressure occurs the neighbouring healthy ones will be over-distended, and will in turn compress and render anæmic the capillaries in the area of the diseased vessel, for the pressure in the former will be higher than in the branches of the occluded vessel beyond the obstruction. Hence a paralysis may occur. If now we give nitrites to abolish the rise of general pressure this effect will cease, and the capillaries of the diseased artery may again become permeable. In other cases the blood pressure may be so high that the heart fails to overcome it and to drive the blood past the obstruction. Here again temporary relief by nitrites may so strengthen the heart that it will succeed in passing on the blood. On either supposition we can account for such cases as I have described. We see that the blood stream is suddenly cut off in a limited area, and paralysis follows. Under favourable conditions the vessels again become permeable, and function is restored. We know of no circulating toxin in many of these patients to cause a spasm, but more or less obstruction can be demonstrated *post-mortem.* Cases illustrating this type of attack and reported by Gowers,[10] Griswold,[11] Fox,[12] Rhein,[13] Grossmann,[14] Davidson,[15] E. O. Daly,[16] Stengel,[17] Edgeworth,[18] Langwill, Lundie and Easterbrook,[19] W. E. Russell,[20] Knapp,[21] Jacobson,[22] and many others show every variety, both in the area affected and in the duration of the paralysis. There is little distinction between the frequency of left and right-sided attacks; sometimes in the same patient the attacks change from one side to the other. There may be gradual or sudden onset and recovery. At times the paralysed patient cries out, "Now I am all right," and the limb is restored at once; or there may be a slow recovery lasting some hours or days. There is nothing distinctive, again, as to convulsions or loss of consciousness. D. J. Macarthy[23] holds that there are two forms of these transient paralyses in arterio-sclerosis. (1) The first is that of cortical exhaustion, directly due to the obstruction cutting off the blood supply temporarily. Here we get the same symptoms as in hemorrhage, namely unconsciousness and paralysis, but temporary only. Similar attacks occur from time to time, till finally softening and permanent effects result. This, by the way, explains a number of cases given by W. E. Russell, where in persons of high tension and sclerotic vessels recurrent attacks were succeeded by a fatal hemiplegia ascribed to hemorrhage. A *post-mortem*, however, showed softening only. (2) The other type is, as he thinks, due to microscopic areas of softening. Here temporary paralyses occur without loss of consciousness. Personally I cannot accept his distinction. He makes, however, a useful suggestion as to the differentiation of these attacks from permanent paralysis at an early stage, in that, on careful watching, slight movements of the affected limb may often be detected in a transient case. We must, however, be careful not to mistake movements in the ingravescent stage of a hemorrhage for such remissions.

An important question arises whether transient paralysis can occur in simple hyperpiesis. We know that high blood pressure exists with perfectly healthy vessels for a time at least in some persons. Can a localised anæmia, as distinct from general syncope, take place here ? I have not yet found an undoubted case on record, and our present knowledge of the brain circulation rather points to its impossibility. We have acknowledged that in Raynaud's disease there is evidence of spasmodic contraction of cerebral vessels as of other vessels, but whether there is a local disease here we do not know. In *migraine*, too, Marcus Gunn notes that hemiparesis and aphasia of a temporary type are sometimes seen. W. E. Russell mentions the case of a young man who when overworked had similar attacks ; and like instances are known to many of us, but in the absence of any knowledge of its pathology explanation is impossible. Osler, indeed, remarks that arterio-sclerosis has been seen on the affected side in some cases.

*Uræmia.*—If we put on one side the paralyses due to cerebral hemorrhage, which are not uncommon in the uræmic state, there are still a remarkable number, often of transient duration, for which no organic cause can be found *post-mortem*. Thus Baillet in thirty-seven instances could find no visible lesions in twenty-nine. Their frequency is a matter of everyday experience. Rhein,[13] indeed, thinks they are due to microscopic areas of softening, which he demonstrated in a few cases; but it is quite possible that these areas are a result, not a cause of the attacks. In several of his instances a syphilitic origin was possible, and at present they do not appear to have been found in a large number of uræmic patients. Others, again, have referred these paralyses to arterio-sclerosis or high blood pressure, both common but by no means universal complications of uræmia. Still, it is quite clear that many cases exist where these causes are absent. R. W. Phillip[24] reports a case of hemiplegia in lardaceous disease where neither hemorrhage nor softening was found *post-mortem*, and no heart trouble or atheroma existed, but only œdema. The theory of uræmic toxins has been widely accepted. Castaignac, believing that the cerebro-spinal fluid in uræmia contains a poison which acts on the nerve tissues directly, injected it into animals, and produced convulsions. Weissenburg,[25] like Rhein, claims to have found changes in the nerve cells and degenerations of the motor tract, but it is difficult to imagine these changes causing transient paralyses. Finally, there has been of late a revival of Traube's old view that they are due to œdema. This may be local or general. Various forms have been given to this theory. Macarthy[26] seems to say that the œdema by compression and obliteration of arteries leads to malnutrition of the cortical cells. We really have to choose between a local cerebral anæmia produced by œdema,[27] (or in some cases by sclerosis) as one explanation; and in opposition to this the theory of the action of uræmic toxins. Now, as Byrom Bramwell[28] points out, toxins are present in large quantities in latent uræmia, and yet produce no symptoms. They are not to any great extent removed by venesection, and yet a slight bleeding will sometimes immediately remove uræmic symptoms. Again, the toxins are clearly carried by the blood stream to all parts of the brain, and one cannot imagine that this affects one side of the brain and not the other, causing the common hemiplegias and monoplegias. Trephining, too, will at times give rapid relief, as in a well-known case of Byrom Bramwell's, and in a more recent one of Cushing and Bordley's.[29] Lumbar puncture has been equally successful in many instances reported by McVail,[30] Willson,[31] and others. Thus in every one of Willson's six cases of uræmic convulsions the movements ceased after the puncture was made. Here the abstraction of a very small amount of fluid sufficed to relieve the pressure ; but the method is not uniformly successful, firstly, because of the ease with which the channel from the cranium is obstructed, and, secondly, because if the œdema is localised and not due to free fluid there can be no drainage. As one of these writers noticed, if fluid flows freely from the trocar the results are much better than when very little comes away. There seems, then, fairly strong evidence that œdema is the cause of many uræmic paralyses, and the results of trephining in the cases referred to are almost conclusive. The other causes suggested appear inadequate, while cerebral anæmia, which is so common a source of transient paralyses, would easily be produced by it. Cerebral œdema in some diseases is said to be merely compensatory to atrophy of nervous tissues, and does not compress vessels ; but there can be no atrophy in acute nephritis, for instance, yet the trephined cases showed a great excess of serum. Cushing and Bordley refer to the close similarity between the retinal changes in many of these cases and those seen in patients where a tumour has interfered with the brain circulation. These retinal changes are also quickly relieved by trephining in either condition. In short, whether œdema is produced by a tumour, a blood-clot, a foreign body, or by the uræmic state, it equally compresses capillary areas. In many other diseases, transient paralyses are also produced by œdema, notably in *meningitis*. Kuh[32] gives a curious instance of recurrent attacks of left hemiplegia in a young man during ten days, each attack lasting about twenty minutes. This was followed by permanent right hemiplegia and death. It must be allowed that there was a history of syphilis, but the arteries were found to be normal. There was no softening or hemorrhage, and merely a great increase of serous fluid in the brain. In *lead*

*encephalopathy* we meet with transient paralyses, as well as other cerebral symptoms. Their distribution is hemiplegic or general, and quite unlike that of ordinary lead poisoning, which picks out certain spinal segments. Oliver[33] points out that the "brain substance may here be pale and very firm, or pale and œdematous as in cases of uræmia." The neuro-retinitis, too, while singularly like that of uræmia, may occur without any affection of the kidneys, and seems not improbably due to the pressure of œdema. Local and temporary œdema, then, is likely to be the cause of these paralyses, but the direct action of lead on the brain substance is also possible. In *eclampsia*[34] and *general paralysis* similar attacks are common. Mott,[3] referring to certain experiments of his own in producing brain œdema, says: "This makes it probable that the epileptiform seizures in general paralysis are dependent on venous stasis, which leads to arterial anæmia and excitability." In diabetes, again, Williamson[36] points out that cases of hemiplegia and other brain affections have been found with no visible lesions to account for them, and he compares them to the paralyses in uræmia. He quotes from Lepine and Blanc an instance of hemiplegia and convulsions in diabetes, with microscopic areas of softening. Œdema of the face or body sometimes occurs, and was noted by Frerichs[37] in 6 per cent. of his cases without kidney disease. Whether my patient's attack was due to cerebral œdema, probable as it is, I have no evidence to show.

In conclusion, a great number of transient paralyses in various diseases appear to be caused by acute localised brain anæmia. This is produced either by vascular degenerations or by compression from active œdema, and accounts for their transitory character.

My grateful thanks are due to Dr. Leonard Hill for kind references and information.

REFERENCES.

1 Leonard Hill, *Physiology of the Cerebral Circulation*, and article in Allbutt's *System*, vol. vii.

2 Dixon Mann, *Forensic Med.* p. 199, ed. 3.

3 *Am. J. M. Sc.*, 1902, cxxiv, 375.

4 *Am. J. Phy.*, 1905, xiv, 452, and 1907, xx, 206, and 1908, xxi, 454.

5 Weiss in Monro's *Treatise on Raynaud's Disease*, p. 151.

[6] *Am. J. M. Sc.*, 1896, cxii, 522.
[7] *Proc. Roy. Soc. Med.*, 1907, i, No. 2, Dec., Med. Sec., p. 116.
[8] W. E. Russell, *Arterial Hypertonus.*
[9] *Am. J. M. Sc.*, 1908, cxxxv, 187.
[10] Gowers, *Diseases of Nervous System*, ii, 348.
[11] *J. Am. M. Ass.*, 1884, iii, 57.
[12] *Brit. M. J.*, 1895, i, 1201.
[13] *J. Am. M. Ass.*, 1906, xlxii, 1705.
[14] *Med. Rec.*, 1902, lvii. 595.
[15] *Liverpool M.-Chir. J.*, 1897, xvii, 433.
[16] *Brain*, 1887, x, 187.
[17] *Am. J. M. Sc.*, 1908, cxxxv. 187.
[18] *Scottish M. and S. J.*, 1907, xix, 414.
[19] *Scottish M. and S. J.*, 1906, xviii, 509, and 1907, xix, 414.
[20] *Loc. cit.*
[21] *J. Nerv. and Ment. Dis.*, 1904, xxxi, 190.
[22] *Deutsche Zeitsch f. Nerv. Heilk.*, 1893–4, p. 235.
[23] *J. Am. M. Ass.*, 1906, ii, 1719.
[24] *Edin. Hosp. Rep.*, 1906, iv, 364.
[25] *Proc. Path. Soc. Phil.*, 1904, vii, 62.
[26] *Internat M. Mag.*, 1902, xi, 514.
[27] *West Lond. M. J.*, 1907, xii, 9.
[28] *Clin. Studies*, 1906, v, 1.
[29] *Am. J. M. Sc.*, 1908, cxxxvi, p. 484.
[30] *Brit. M. J.*, 1903, ii, 1054.
[31] *J. Am. M. Ass.*, 1904, xliii, 1019.
[32] *J. Nerv. and Ment. Dis.*, 1905, xxxii, 471.
[33] Article in Allbutt's *System*, ii.
[34] Slemmons, *Johns Hosp. Bull.*, 1907, p. 448.
[35] Mott, *Arch. of Neurol.*, v, 500.
[36] Williamson, *Diabetes Mellitus.*
[37] F. T. Frerichs, *Ueber den Diabetes.*

